# Primary and Cutaneous Lesions in the Male

**Published:** 1920-04-24

**Authors:** 


### An Atlas of Syphilis.

### Primary and Cutaneous Lesions in the Male.

We draw particular attention to this recently-published volume* for two distinct and separate reasons. The first is that war has brought the question of venereal disease to the front rank amongst social problems of national importance, and in the medical profession's combat with this evil it is absolutely necessary that the best modern treatment be undertaken at the earliest possible moment after infection has occurred. The second is that a really well-produced photographic atlas of syphilitic lesions, as they may be met, has been badly needed to supplement and aid the various more elaborate textbooks which deal exhaustively with the clinical side of the subject. The authors have made great and valuable use of stereoscopic photography, a method of bringing out the actual appearance of pathological conditions which is strongly to be recommended.

Illustrative Value.

Although depending chiefly upon its illustrative value, this work includes a short description of each of the common types of syphilitic cutaneous lesion, and concludes with a singularly useful section, Part VI., in which some common skin diseases not infrequently mistaken for syphilis are brought to the reader's notice, both pictorially and in the text. The cases from which this excellent choice was made were met with at a large military venereal hospital, where some 19,000 cases of syphilis came under observation, and, fortunately, although not originally contemplating publication, the authors made it a routine practice to photograph selected cases. The result of their labours well merits inspection by any medical men who are likely to be called upon to deal with venereal cases.

* *An Atlas of the Primary and Cutaneous Lesions of Acquired Syphilis in the Male.* By Major Charles F. White and Dr. W. Herbert Brown. John Bale, Sons and Danielsson, Ltd., 91 Great Titchfield Street, London, W. 1. Price 27s. 6d. net.

